# Understanding the role of dynamics in the iron sulfur cluster molecular machine

**DOI:** 10.1016/j.bbagen.2016.07.020

**Published:** 2017-01

**Authors:** Danilo di Maio, Balasubramanian Chandramouli, Robert Yan, Giuseppe Brancato, Annalisa Pastore

**Affiliations:** a Scuola Normale Superiore, Piazza dei Cavalieri 7, I-56126 Pisa, Italy; b Istituto Nazionale di Fisica Nucleare (INFN) sezione di Pisa, Largo Bruno Pontecorvo 3, 56127 Pisa, Italy; c Department of Neuroscience, Wohl Institute, King's College London, Denmark Hill Campus, London SE5, UK; d Immunologia Patologia Generale Department, University of Pavia, Via Ferrata, 9, 27100 Pavia, Italy

**Keywords:** NMR, nuclear magnetic resonance, MD, molecular dynamics, PCA, principal component analysis, ED, essential dynamics, RMSD, root-mean square deviation, RMSF, root-mean-square fluctuation, CyaY, Frataxin, Iron-sulfur cluster biogenesis, Molecular dynamics, Structure

## Abstract

**Background:**

The bacterial proteins IscS, IscU and CyaY, the bacterial orthologue of frataxin, play an essential role in the biological machine that assembles the prosthetic Fe—S cluster groups on proteins. They form functionally binary and ternary complexes both in vivo and in vitro. Yet, the mechanism by which they work remains unclear.

**Methods:**

We carried out extensive molecular dynamics simulations to understand the nature of their interactions and the role of dynamics starting from the crystal structure of a IscS-IscU complex and the experimentally-based model of a ternary IscS-IscU-CyaY complex and used nuclear magnetic resonance to experimentally test the interface.

**Results:**

We show that, while being firmly anchored to IscS, IscU has a pivotal motion around the interface. Our results also describe how the catalytic loop of IscS can flip conformation to allow Fe—S cluster assembly. This motion is hampered in the ternary complex explaining its inhibitory properties in cluster formation.

**Conclusions:**

We conclude that the observed ‘fluid’ IscS-IscU interface provides the binary complex with a functional adaptability exploited in partner recognition and unravels the molecular determinants of the reported inhibitory action of CyaY in the IscS-IscU-CyaY complex explained in terms of the hampering effect on specific IscU-IscS movements.

**General significance:**

Our study provides the first mechanistic basis to explain how the IscS-IscU complex selects its binding partners and supports the inhibitory role of CyaY in the ternary complex.

## Highlights

• IscU protomers show a pivotal motion around the interface with IscS, in complex.
• Both IscS catalytic loop and IscU motion seem correlated with Fe—S cluster assembly.
• Shuttling of the IscS catalytic loop is accompanied by a structural transition.
• CyaY inhibits cluster formation by hampering the dynamics of the IscS-IscU complex.

## Graphical abstract

**Figure:**
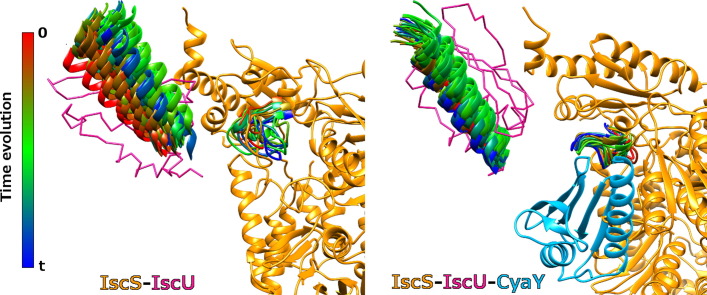

## 1 Introduction

The bacterial proteins IscS and IscU are essential cellular components which take part in iron-sulfur cluster biogenesis [1], [2]. Iron-sulfur clusters are evolutionary ancient prosthetic groups found in several different pathways where they provide their unique redox potential. IscS is the desulfurase which converts cysteine to alanine and forms highly reactive persulfide species. It is not only involved in iron-sulfur cluster formation as it is the enzyme that provides sulfur also in the molybdenum cofactor (Moco) biosynthesis and tRNA thiolation [3], [4], [5]. IscU is the scaffold protein which hosts transiently the iron-sulfur cluster and eventually delivers it to the final acceptors. IscS and IscU form a hetero-tetrameric complex together and are highly conserved from bacteria to primates, with the eukaryotic orthologues being known as Nfs1 and Isu1, respectively.

Several structures of these two proteins are available, both in isolation and in a complex [6], [7], [8]. From them, we have learned that, when in the test tube and not in a complex, IscU can be in at least two conformational states, one compactly folded (S state), the other partially unfolded (D state), where the N-terminal helix is detached from the rest of the structure and highly flexible in solution [9]. Free IscU is an intrinsically unstable protein able to undergo both cold and heat denaturation at detectable temperatures [10], [11]. However, such flexibility is not observed in the crystal structures of IscS-IscU complexes or in solution when bound to IscS or to zinc [6], [7], [8], [11], [12]. In these complexes, IscU appears fully folded, putting in doubt the physiological importance of the D state. It is also possible that, also in the absence of a binding partner, crowding stabilizes the protein in the cell to its compact form.

In the IscS-IscU complex, the obligate IscS dimer interacts with two copies of IscU. Each copy sits close but not in direct contact with the catalytic site of IscS and binds the roughly prolate ellipsoidal IscS dimer at the opposite extremes of the two poles, far away from each other. The active site of IscS contains a covalently bound pyridoxal phosphate (PLP) group, a catalytic cysteine (C328 in the *E. coli* structure numbering) hosted in a flexible loop, and a lysine (K206). During catalytic activity aimed at the production of iron-sulfur clusters, the loop is thought to move from the active site to IscU to deliver the persulfide which willl form the cluster together with iron. Additionally, IscS forms complexes with several other partners, such as the proteins TusA [8], ferredoxin [13], [14], CyaY [15], and the ancillary protein YfhJ (also known as IscX) [8], [16], which all belong to the machine that is responsible for iron-sulfur cluster biogenesis. Of these, only the structure of the TusA-IscS complex has been determined at high resolution [8]. Experimentally-based models exist for the complexes with frataxin, ferredoxin and YfhJ [14], [15], [17]. Interestingly, all these proteins seem to compete for the same surface of IscS, which is close to the active site but distinct from the region which hosts IscU [8], [14], [15].

Among these interactions, a particularly interesting one is that with CyaY, since this protein is the bacterial ortholog of frataxin which, in humans, is linked to the neurodegenerative Friedreich's ataxia (MIM 229300). This disease is caused by reduced levels of frataxin [18]. Previous studies have shown that frataxin intervenes in regulating the speed of iron-sulfur cluster formation, although, paradoxically, in prokaryotes appears to act as an inhibitor while in eukaryotes is an activator [19].

Several mechanistic questions remain open about the IscS-IscU and IscS-IscU-CyaY complexes. We still do not know, for instance, what part the dynamical behaviour of IscU and its intrinsic flexibility plays in complex formation with IscS. How the catalytic loop of IscS bearing the persulfide group moves from the catalytic site to the acceptor (i.e., IscU) and what is the role of CyaY in such movements are other important aspects that need elucidation. In one of the crystal structures of the complex, the catalytic loop is so flexible not to be observable [8]. This is not the case for the structure of the IscS-IscU complex from *A*. *fulgidus* where the loop is close to IscU providing a potential alternative ligand for the cluster [6], [7]. Only a low resolution model of the ternary IscS-IscU-CyaY complex is available, hampering direct evaluation of how its presence close to the catalytic loop might affect it. Finally, it is also unclear what is the pathway followed by iron to reach IscU [15].

To address these open questions and to provide more insights into the dynamics of the IscS-IscU complex, both in the presence and in the absence of CyaY, we have carried out an extensive study based on molecular dynamics (MD) simulations and experimental validation. We show that the binary IscS-IscU complex is stably folded and features a likely functionally relevant pivotal motion of IscU around the interface with IscS. We also show how the mobility of the catalytic loop of IscS is strongly reduced by the presence of CyaY and suggest the mechanism by which this protein stabilizes the IscS-IscU interaction and acts as an inhibitor. We validated experimentally, using nuclear magnetic resonance (NMR), the nature of the IscS-IscU interface and found that, despite the tight anchoring of IscU, the side chains of some groups are trapped in different conformations according to what we can predict from our simulations. Overall, our findings bear important consequences for understanding the role of frataxin in iron-sulfur cluster biogenesis.

## 2 Materials and methods

### 2.1 IscS-IscU complex model

The *E*. *coli* IscS-IscU complex (PDB code: 3LVL) [8] was used for molecular dynamics study of the binary complex. A missing segment of the IscS loop bearing the catalytic cysteine (residues 328–334) was reconstructed using the Modeller [20] module (v 9.12) as implemented into the UCSF-Chimera software package [21]. Five different conformations of the loop were generated. We selected as the starting conformation the one where the loop was most equidistant from both IscS and IscU so as not to artificially orient the dynamics of the loop towards one side or another of the binding interface. The same coordinates were applied to both missing loop segments to avoid possible inconsistencies. The complex was then solvated in a ~ 23 Å layer cubic water box using the TIP3P water model parameters. Na^+^ and Cl^−^ ions were added to ensure system electroneutrality and to set the final concentration to 0.15 M. The final system size was 188 Å × 95 Å × 105 Å for a total number of atoms of ~ 170,000. The system was minimized in two stages: first, a 20,000-step run was carried out with restraints on all the protein atoms (5 kcal/mol/Å^2^). A further 10,000-step minimization was carried out by applying the restraints on the cofactor and C_α_ protein atoms only. A short (200 ps) NPT simulation at 200 K and 1 atm was performed with restraints on all the protein atoms (5 kcal/mol/Å^2^), to adjust the volume of the simulation box, while preserving the minimized protein structure obtained in the previous steps. The system was slowly heated up to 300 K over a 3 ns period, again applying the restraints on the cofactor and C_α_ atoms only and gradually releasing them to 1 kcal/mol/Å^2^ along the thermalization process. Subsequently, the system was equilibrated for 2 ns, gradually reducing the restraints to zero.

### 2.2 IscS-IscU-CyaY complex model

The *E*. *coli* IscS-IscU-CyaY complex structure [15] was used as the starting point for the molecular dynamics of the ternary complex. The procedure to prepare the system was slightly different to minimize the steric clashes between the CyaY monomers and the IscS-IscU subunits. First, the side chains of the residues at the interface between the CyaY monomers and the corresponding IscS-IscU subunits were manually adjusted by means of the UCSF-Chimera software package [21]. The simulation box was prepared as for the IscS-IscU complex. The final system size was 194 Å × 102 Å × 111 Å for a total number of atoms of ~ 205,000. The system was then minimized and equilibrated in the following fashion. A first round of 15,000-step energy minimization was performed with restraints on the C_α_ protein atoms and on the cofactor atoms (5 kcal/mol/Å^2^), followed by a subsequent 20,000-step energy minimization run with no restraints to minimize as much as possible steric clashes in the first stage. A short (200 ps) NPT simulation at 200 K and 1 atm was performed restraining all atoms (5 kcal/mol/Å^2^) to adjust the volume of the simulation box, while preserving the minimized protein structure obtained in the previous steps. Afterwards, the system was slowly heated up to 300 K over a 3 ns period, again applying the restraints on the cofactor and C_α_ atoms only and gradually releasing them to 1 kcal/mol/Å^2^ along the thermalization process and finally equilibrated for 6 ns, gradually reducing the restraints to zero.

### 2.3 Molecular dynamics simulation and analysis

Production runs were performed under NPT conditions at 1 atm and 300 K and extended up to 400 ns for both complexes. A 10 Å cutoff (switched at 8.0 Å) was used for atom pair interactions. The long-range electrostatic interactions were computed by means of the particle mesh Ewald (PME) method using a 1.0 Å grid spacing in periodic boundary conditions. The RATTLE algorithm [22] was applied to constrain bonds involving hydrogen atoms, and thus a 2 fs time step could be used. All the systems were simulated with NAMD [23] (v. 2.9), using the ff99SBildn Amber force field parameters [24], [25] for protein and ions. The parameters for PLP were generated in two steps. Initially, charges were computed using the restrained electrostatic potential (RESP) fitting procedure [26]. The ESP was calculated by means of the Gaussian09 package [27] using a B3LYP/6-31G* level of theory. The RESP charges were obtained by a two-stage fitting procedure using the program RED [28], [29]. Missing bond, angle, torsion and improper torsion angle parameters were generated using Antechamber [30]. Analyses were performed using the cpptraj [31] tool and in-house scripts exploiting the MDAnalysis library [32]. Principal Component Analysis (PCA) was performed by means of the Gromacs analysis tools [33] g_covar and g_anaeig, restricting the analysis to the Cα atoms only. The electrostatic potential was evaluated using the APBS server [34]. Plots were created using the Matplotlib software [35]. Figures were generated using the UCSF-Chimera software package [21] and the VMD program [36].

### 2.4 Sample preparation and NMR experiments

*E*. *coli* zinc-bound IscU (Zn-IscU), IscS and CyaY were prepared as described elsewhere [37], [38]. U-[15N, 2H], Ileδ1-[13C, 1H], Leuδ1/2-[13C, 1H], Valγ1/2-[13C, 1H] samples (henceforth referred to as U-[15N, 2H], ILV^CH3^) and U-[2H] were prepared in M9 minimal media supplemented where appropriate with 99% D_2_O (2H, 99%; CIL), ammonium sulphate (15N_2_, 99%; CIL), 2H d-glucose (1,2,3,4,5,6,6-D_7_, 98%; CIL) with further addition 1 h before induction with the ile, leu and val precursors α-ketobutyric acid (3-Methyl-13C, 99%; 3,3-D_2_, 98%; CIL) and α-ketoisovaleric acid (3-Methyl-13C, 99%; 3,4,4,4-D_4_, 98%; CIL) [39]. Samples for NMR were prepared in 20 mM Tris-HCl at pH 8, in 150 mM NaCl, 5 mM TCEP, 0.05% NaN_3_ and 100% D_2_O. IscU-IscS titrations were observed by NMR using methyl TROSY optimised HMQC spectra [40] recorded on an Avance III Bruker 800 MHz spectrometer with TXI cryoprobe at the MRC Biomedical NMR Centre.

## 3 Results

### 3.1 Stability and structural fluctuations of the IscS-IscU complex

**Fig. 1:**
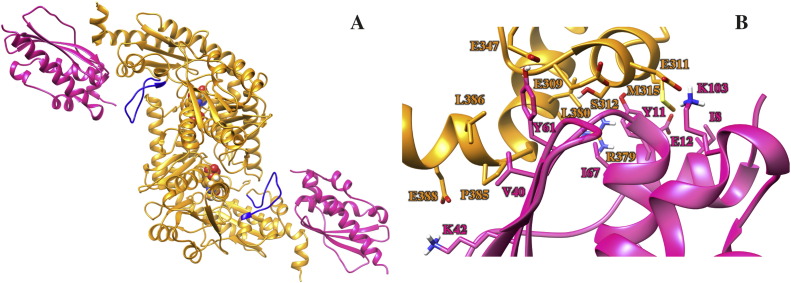
**The IscS**-**IscU complex**. (A) The complex structure, as formed by the IscS dimer (orange) and two IscU protomers (pink) as obtained starting from the 3LVL coordinates. The catalytic loop of IscS is colored in blue and the PLP cofactor is displayed as spheres. (B) Zoomed view of the interface between IscS and IscU. Side chains of the main interacting residues are shown as sticks, while the rest of the protein is depicted as cartoons.

The *E*. *coli* IscS-IscU complex was simulated in aqueous solution at room temperature for 0.4 μs. The overall structure of the complex remains basically unchanged throughout this time interval (Fig. 1A), showing no sign of major unfolding or rearrangements. The N-terminus of IscU remains structured in the simulated complex, adopting a helical conformation (Fig. S1) as observed in all crystal structures where it is in complex with IscS. This supports the hypothesis that IscU unfolding and thus the D state may occur more easily when the protein is not in a complex with IscS and/or zinc [9].

IscU is firmly anchored to IscS through the interface shaped by IscU residues I8, Y11, E12, V40, K42, Y61, I67 and K103 and IscS residues E309, E311, S312, M315, E347, R379, L380, P385, L386 and E388 (Fig. 1B), according to the crystal structure numbering. K42, Y61 and K103 of IscU form salt bridges or hydrogen bonds with E388, E347 and E311 of IscS, whereas the other residues mostly form apolar interactions with each other or are involved in other non-specific contacts with either IscS or IscU. Despite such a stable binding, IscU undergoes a gentle rocking motion with respect to IscS around its anchoring interface. This motion results in the opening and closing of a cavity (‘catalytic mouth’) formed between the IscS dimer interface and IscU, which hosts the catalytic site as well as CyaY and other IscS partners. The pivotal motion could thus be important in the mechanism of recognition and partner selection (vide infra).

**Fig. 2:**
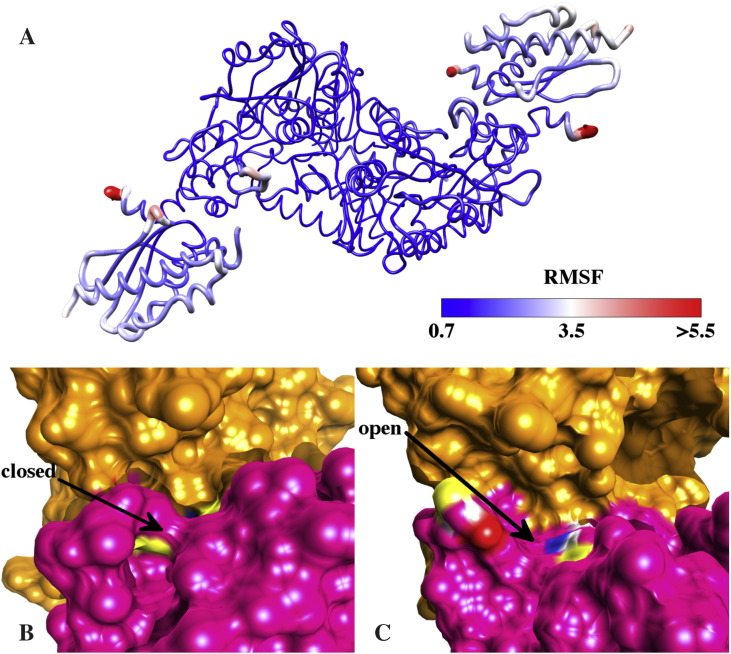
**Backbone RMSF and IscU***β*-**turn opening motion**. (A) Average structure of the IscS-IscU tetramer and E. coli IscS-IscU crystal structure (PDB ID: 3LVL) from the binary complex simulation, depicted as ribbons and colored according to backbone RMSF values, from lowest (blue) to highest (red). Ribbon thickness is proportional to the local RMSF value. Detailed view of the IscU active site in the binary complex showing a (B) closed form of its *β*-turn from the *E*. *coli* IscS-IscU crystal structure (ID PDB: 3LVL) and (C) an open form of the same turn from the average IscS-IscU complex structure from our simulation. In both B and C panels, IscS (orange) and IscU (pink) are displayed as surfaces.

Analysis of the structural fluctuations of the complex through the evaluation of the root mean square fluctuations (RMSF) reveals three main flexible regions of such a hetero-tetramer (Fig. 2A). The first is represented by the C-termini of the two IscS protomers. The second is a small *β*-turn (i.e. residues 34–39) in IscU, which contains one of the three conserved cysteines (i.e. C37) involved in Fe—S cluster coordination. Interestingly, we observe an opening motion of this *β*-turn in one of the two IscU protomers, leading to an increased exposure of the “active site” of IscU (Figs. 2B, 2C). The last flexible region contains the catalytic loop of IscS (i.e. residues 323–337). Our analysis also shows relatively high structural fluctuations distributed across several secondary structure elements of IscU, particularly the α-helices but also a few *β*-turns and *β*-sheets which comprise residues 21–28, 41–56, 64–87 and 108–126. These regions mostly involve an exposed region of IscU far from the binding interface.

### 3.2 Dynamics of the IscS catalytic loop

The catalytic C328 of IscS is in a loop (i.e. residues 323–337) which is believed to be, upon cysteine desulfuration, the carrier of a persulfide group from the PLP group in the catalytic site to the relatively distant (~ 15 Å) IscU active site. We thus analysed the dynamics of the loop in detail. We monitored three different parameters to identify structural changes possibly correlated with the transport mechanism: 1) the secondary structure evolution of the loop, 2) its radius of gyration and 3) the distance between the cysteine sulfur atom of C328 and those belonging to C63 and C106 in the IscU active site (C37 omitted due to the flexibility of the *β*-turn in which it resides).

**Fig. 3:**
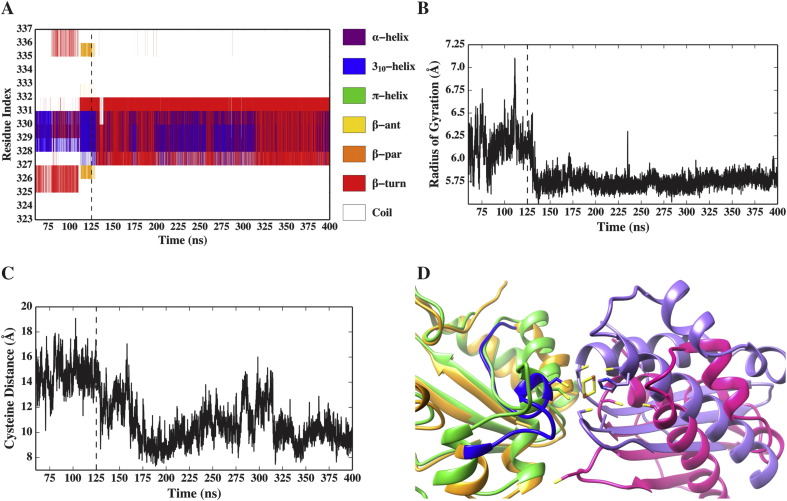
**Conformational transition of the IscS catalytic loop**. Time evolution of the conformational change of the IscS catalytic loop, as monitored by (A) secondary structure, (B) radius of gyration (only backbone atoms) and (C) distance between C328 and the IscU active site (i.e. C63 and C106). (D) Structural superposition of a representative configuration of the IscS (orange)-IscU (pink) complex (catalytic loop in blue), extracted from the MD trajectory at ca. 225 ns, and the *A*. *fulgidus* IscS (green)-IscU (purple) crystal structure (only IscS was used for the fitting). C328 and the conserved cysteines on IscU are shown as sticks, as well as the [Fe—S] cluster in the *A*. *fulgidus* structure. Note the structural transition at about 125 ns (panel A–B) triggering a progressive approach of C328 towards the active site of IscU (panel C).

Our simulation shows a structural transition in the IscS loop. After about 125 ns, a small portion of the loop (i.e. residues 328–332) undergoes a structural rearrangement from a mostly 3_10_-helical structure to a conformational state characterized by a *β*-turn/3_10_-helix equilibrium, which is maintained throughout the rest of the simulation (Fig. 3A). At the same time, the radius of gyration of the loop decreases to a more compact structure (Fig. 3B). This conformational transition, which is likely triggered by the breaking of an adjacent *β*-turn (i.e. residues 325–327), enables a progressive approach of C328 towards the IscU active site, up to a distance of ~ 9 Å (Fig. 3C). It is worth noting that the latter distance is relatively close to that observed in the X-ray structure of the *A*. *fulgidus* IscS-IscU complex (PDB code 4EB5) (~ 6 Å), in which the Fe—S cluster is assembled and bound to the loop cysteine [6]. Superposition of the IscS loop from our simulation and the holo crystal structure displays remarkable similarities (Fig. 3D).

These observations suggest that movement of the catalytic loop requires subtle conformational rearrangements of IscS. In the next section, further analyses focus on global and correlated motions of the protein complex potentially related with cluster biosynthesis.

### 3.3 Collective motion analysis of the IscS-IscU complex

**Fig. 4:**
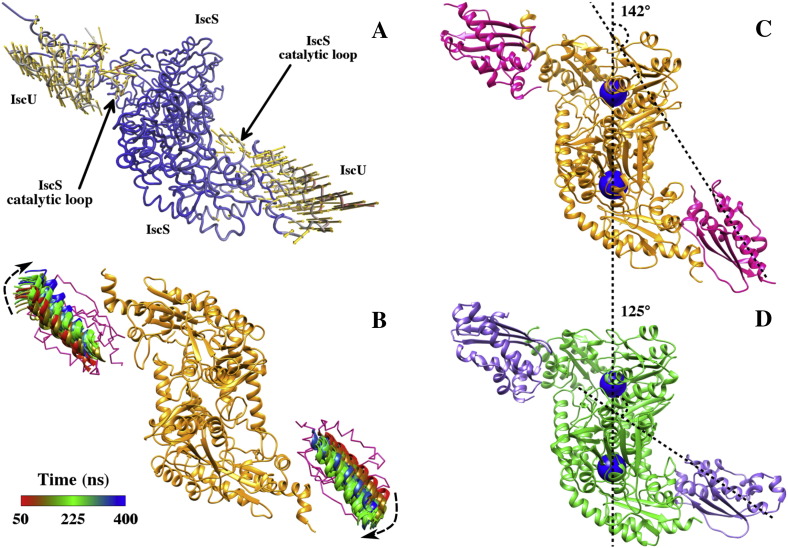
**IscU orientation and dynamics within the binary complex**. (A) Main collective motions of the IscS-IscU complex from PCA (Cα atoms only), showing the IscU pivotal movement around the IscS dimer and the motion of the IscS catalytic loop. Vectors display the amount and direction of the residue motion along the first eigenvector. Minor residue contributions (i.e. arrow length < 2Å) were omitted. (B) Motion of the IscU protomers around the IscS dimer. A number of uncorrelated IscU configurations (one every 10 ns) are superposed upon fitting onto the IscS dimer structure (orange). A representative α-helix of IscU is depicted as cartoons and colored as a function of simulation time, while the rest of the protein is depicted as pink ribbons. Angle formed between IscU and IscS from (C) the average IscS-IscU complex structure from our MD simulation, and (D) the *A*. *fulgidus* crystal structure.

Motivated by the observed pivotal motion of IscU around the IscS interface, we performed a principal component analysis (PCA) of the binary complex dynamics, aiming at identifying the most significant collective motions. The analysis clearly shows a major contribution of the two IscU monomers to the first large amplitude motion of the complex (Fig. 4A), in addition to the IscS catalytic loops and the IscU *β*-turns. This is in agreement with the motions previously evidenced by the structural fluctuation analysis. A closer inspection of the collective motions described by the first PCA eigenvector (> 50% of the total variance of the system) points towards a coherent rocking motion of IscU as a whole with respect to the interface with the IscS dimer (Fig. 4B). Similar indications emerged also from the analysis of the second and third eigenvectors (data not shown). The PCA results are also consistent with the dynamical picture of the IscU motion based on an intuitive geometrical parameter (i.e. *θ* angle), as reported in the previous section.

To further characterize the pivotal motion suggested by PCA, we estimated the residue-wise dynamic cross correlations (DCC) (Fig. S2). The DCC map emphasizes several significant correlations (*R* ≥ 0.5) among residues belonging to the same IscU protomer using an intensity-based colour code. Most of the off-diagonal correlations (i.e. non-self, *R* ≥ 0.75, colored in red and brown) cluster among the secondary structure elements already identified as the most flexible regions and on the external tip of IscU, which is not involved in IscS binding.

Interestingly, in the structure of the archaebacterium *A. fulgidus* IscS-IscU complex (PDB code 4EB5) [6], where CyaY or frataxin othologues are absent, the ‘catalytic mouth’ is more “closed”, with IscU closer and bent towards the dimer interface. As a result, the geometrical angle, *θ*, formed between the axis on which the centers of mass of both IscS monomers lie and a representative *α*-helical axis of IscU (residues 100–120) is 125° in the *A. fulgidus* crystal structure (Fig. 4D), whereas it spans a rather wide interval throughout the trajectory (i.e. 123°–161°; on average *θ* = 142°) (Fig. 4C).

Altogether these findings show that the IscS-IscU interface retains a certain degree of flexibility and forms a “molecular junction”, despite the stability of the secondary and tertiary structures of the individual components. These motions could favour partner recognition and play a role in favouring the disassembly of IscU from IscS once the Fe—S cluster is formed.

### 3.4 The role of electrostatics in CyaY recognition

**Fig. 5:**
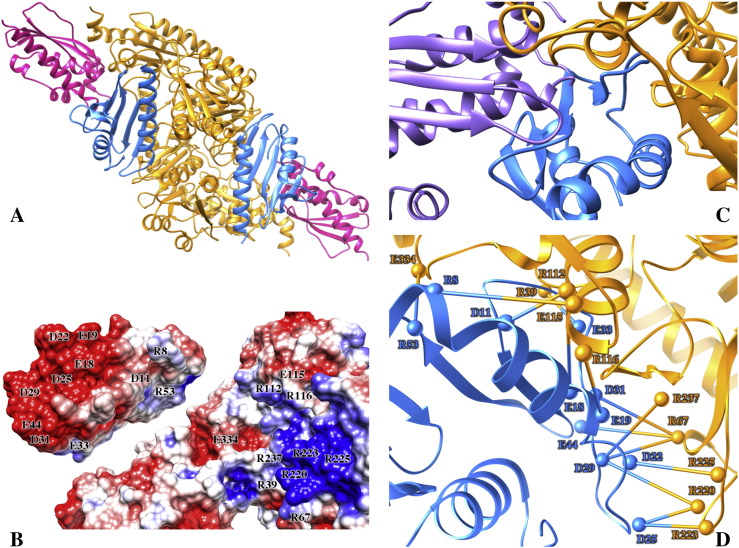
**The IscS**-**IscU**-**CyaY complex**. (A) IscS-IscU-CyaY complex structure, where two CyaY proteins (blue) are bound in the cleft formed between IscS dimer (orange) and IscU (pink). (B) Electrostatic potential surfaces for the IscS-IscU complex (right) and CyaY (left). Structure orientation is chosen to better visualize the mutual interacting surfaces on both systems. (C) Steric clashes between CyaY and [Fe—S]-loaded IscU (purple) in a hypothetical ternary complex structure built from the superposition of *A*. *fulgidus* IscU onto the average ternary structure (with IscU omitted) from our simulation superposed on the IscS dimer only. (D) Zoomed view of the interface between the IscS dimer and CyaY. The more persistent intermolecular salt bridges (see Table S1) are depicted as sticks connecting Cα atoms (spheres) belonging to the corresponding charged residues.

For comparison, we carried out a MD simulation of the IscS-IscU-CyaY ternary complex (Fig. 5A), starting from the coordinates of a model previously obtained via an experimentally-guided protein-protein docking [15]. The original model was based on evidence from NMR detection of the IscS-CyaY interface, calorimetry and SAXS data, and was validated by mutagenesis studies. Analysis of the electrostatic potential calculated for the entire IscS-IscU complex surface shows an overall negative potential, with a few significant exceptions, and includes the R220, R223 and R225 arginine triplet of IscS (Fig. 5B). Mutation of these residues abolishes binding of IscS with several partners, among which CyaY, Fdx and YfhJ [14], [15], [16].

The IscS-IscU-CyaY complex remained stable throughout the simulation (0.4 microseconds) (Fig. S3). CyaY is partially inserted in the ‘catalytic mouth’ delimited by the IscS dimer interface and the IscU binding site (Fig. 5A). The presence of CyaY is sterically incompatible with small angles as that observed in the *A. fulgidus* structure (Figs. 5C) confirming that IscU orientation may impact on partner sensitivity. The size of the cleft strongly depends on the *θ* angle described above. In our simulations, CyaY sits close to the catalytic loop of IscS, making further contacts with residues E334, S332, S330, T329 and A327 on the loop via residues located on the *β*-sheets (namely, N35, V38, N52, R53) and far from the PLP group (~ 18 Å). In addition to the previously identified arginine triplet, interaction of CyaY with the IscS dimer involves an extended network of salt bridges, among which the IscS residues R39, R67, R112, R116, R220, R223, R225 and R237 with CyaY D11, E18, E19, D22, D25, D29, D31, E33, E44 residues. There are also salt bridge pairs between IscS E115 and E334 and CyaY R8 and R53, respectively, indicating further electrostatic components in the interface interaction (Table S1 and Fig. 5D). The CyaY *β*-sheet also weakly interacts with IscU mostly through hydrophobic residues, involving the exposed W61 residue of CyaY and C37, P35, A36, P101, V102 and I104 of IscU. This is in agreement with the high conservation of W61.

### 3.5 The effect of CyaY on the IscS-IscU junction

**Fig. 6:**
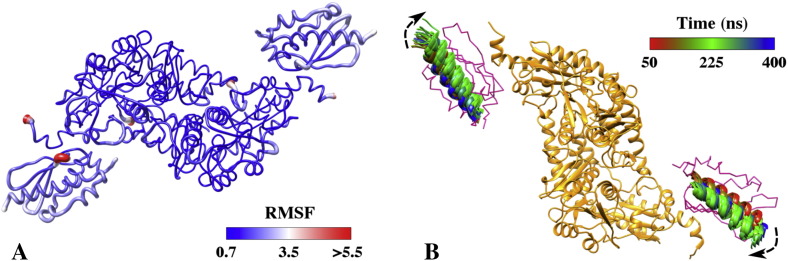
Backbone RMSF and IscU motion within the ternary complex. (A) Average structure of the IscS-IscU hetero-tetramer from the ternary complex simulation, depicted as ribbons and colored according to backbone RMSF values, from lowest (blue) to highest (red). Ribbon thickness is proportional to RMSF value. (B) IscU protomer motions around the IscS dimer from the ternary complex simulation. Details described in the caption of Fig. 4D.

Analysis of the fluctuations of the IscS-IscU tetramer in the ternary complex highlights a significant reduction of the IscS-IscU motions upon CyaY binding (Fig. 6A). The only residues displaying some degree of structural fluctuation are located on the IscU and IscS termini and on the IscU *β*-turn mentioned earlier. IscU, as a whole, retains some degree of flexibility in the ternary complex, though to a lesser extent than in the binary complex (Fig. 6B). These findings are in agreement and explain the tighter affinity (i.e. two orders of magnitude) of the IscS-IscU complex, when CyaY is also bound [15]. The catalytic loop of IscS holds tightly against CyaY and does not display relevant motions towards or away from IscU.

**Fig. 7:**
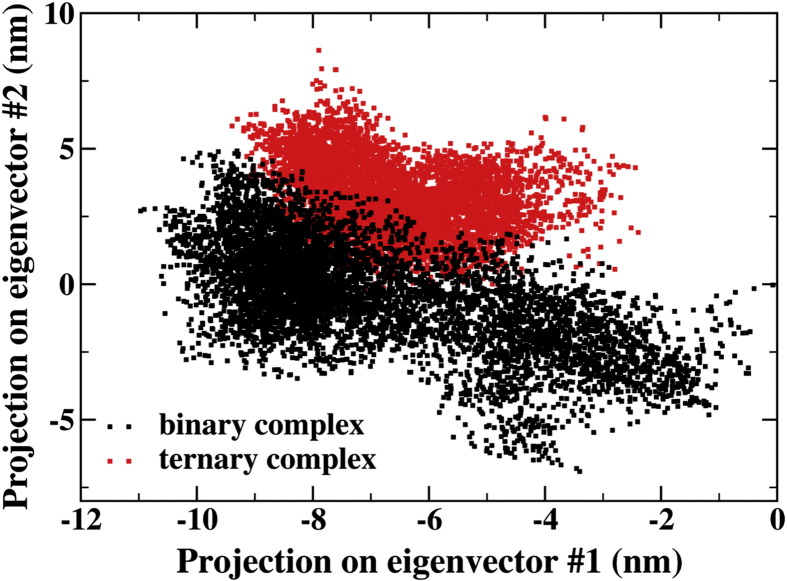
**Essential dynamics analysis of the binary and ternary complexes**. Projection of the simulated trajectories of the IscS-IscU tetramer (Cα atoms only) for both binary and ternary complexes onto the 2D ED plane described by the first two eigenvectors of the binary complex. The analysis highlights the more restricted conformational subspace spanned by the IscS-IscU assembly within the ternary complex with respect to the binary system.

Further differences can be highlighted through essential dynamics (ED) analysis of the simulated trajectories in the presence and the absence of CyaY (see Supplementary Information for more details). The essential 2D subspace spanned by the IscS-IscU system is remarkably different in the two cases, showing only a poor superposition and thus supporting an altered dynamics of the large amplitude motions. A narrower conformational space spanned by the IscS-IscU-CyaY system as observed in the MD projection onto a bidimensional ED plane (Fig. 7) does support a noticeable hampering effect of CyaY on the IscS-IscU dynamics. These conclusions are also supported by a detailed per-residue analysis of the first ED eigenvector (Fig. S4).

### 3.6 The IscS-IscU interface is “fluid”

**Fig. 8:**
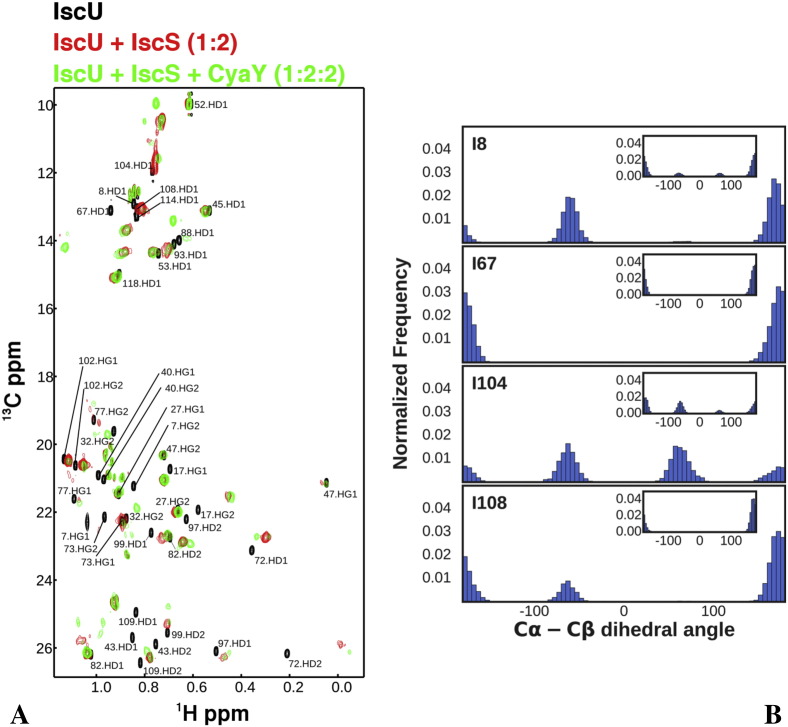
**NMR TROSY spectra and Ile side**-**chain conformational distributions**. (A) Methyl TROSY spectra of 100 μM U-[15N, 2H], ILV^CH3^ IscU-Zn (black spectrum) was titrated with 2 molar equivalences of U-[2H] IscS (red spectrum) and then with 2 molar equivalences of U-[2H] CyaY (green spectrum). The assignments of the Ileδ1, Leuδ1/2 and Valγ1/2 for free IscU-Zn are shown. (B) Distributions of the Cα-Cβ dihedral angle of selected IscU isoleucines from the binary system simulation. Each plot reflects the conformation of both isoleucines of the two protomers.

One important aspect which emerged from our simulations is that, while the backbones of IscS and IscU remain mainly unchanged, the side chain conformation of residues in the interface are compatible with different local minima (Fig. 8A). If experimentally supported, the concept of interface “fluidity” could create a new paradigm in molecular recognition, where the side chains of an interface adopt multiple conformations while the backbone remains tightly attached. To explore experimentally this concept, we recorded methyl TROSY experiments on a selectively labelled sample of IscU. This NMR experiment allows direct detection of selectively labelled isoleucine and leucine side chains in large complexes such as those formed by IscS-IscU (110 kDa) and IscS-IscU-CyaY (130 kDa), while deuterating the molecule to reduce the effective number of spins and thus reducing T_2_ relaxation. Particularly diagnostic of an interface are the CδΗ_3_ side chains of isoleucines which have very distinct chemical shifts in the NMR spectrum around 14 ppm and 0.8 ppm in the 13C and 1H dimension, respectively, and do not overlap with any other residue. IscU contains 11 isoleucines. In isolated IscU, we observe the correct number of resonances (Fig. 8B). By forming the IscS-IscU complex using a 1:2 molar ratio, we observe instead at least 14 resonances. Furthermore, the number of isoleucine resonances increase to 18 when also CyaY is present. Likewise, the number of resonances corresponding to leucines and valines which is expected to be 29, is 31 in the IscS-IscU complex and 37 in the ternary complex. The additional resonances could tentatively be ascribed to different conformations of I67 and I8 and V17, V31 and V40 which are in the interface. These findings testify the presence of a complex equilibrium between species at the interaction interface between the two proteins and tell us that interfaces can often be much more complex than usually assumed even for proteins tightly anchored to each other.

## 4 Discussion and conclusions

In this work, we describe an in-silico study of the IscS-IscU and IscS-IscU-CyaY complexes which was carried out with the aim of extracting new mechanistic information from previous high and low resolution experimental models of these complexes. Our results indicate that, while firmly assembled, these complexes have interesting and specific dynamical features, which may play an important role in their functions. We could, for instance, follow the dynamics of the catalytic loop of IscS which has been previously proposed as the sulfur carrier shuttling from the catalytic site, where it forms a persulfide from the conversion of cysteine to alanine, to the Fe—S cluster biosynthesis site on IscU [6]. In agreement with this hypothesis, we observed for the first time the spontaneous, albeit partial, shuttling of the loop between the two very distant active sites. Our simulation suggests that such a motion is accompanied by a loop structural transition leading to a more compact and structured conformation. After this transition occurs, the loop assumes a conformation close to that observed in a crystal structure where the Fe—S cluster is fully formed.

Despite previous suggestions of a structural flexibility of isolated IscU in solution [41], its structure remains stably folded throughout our simulations, showing no significant deviations from the fold observed in various crystal structures representing different functional states, i.e. bound to IscS both in the absence (PDB code: 3LVL) [8] and in the presence of the Fe—S cluster (PDB code: 4EB5) [6] or in a homotrimeric complex which hosts the cluster (PDB code: 2Z7E) [42]. On the other hand, our analyses clearly indicate that IscU retains some degree of flexibility upon complex formation, providing evidence for a pivotal motion of each IscU monomer independently with respect to the IscS dimer. We believe that such a plasticity may well correlate with the relatively weak affinity of the two proteins (Kd of the IscS-IscU complex is 1 μM) [15]. Loose binding of IscU to IscS is also consistent with the biological role and the biochemical properties of both proteins, which need to detach to allow further transfer of the cluster to other recipient proteins. Some degree of adaptability of the complex quaternary structure may also allow the accommodation of other proteins that play a role in the Fe—S cluster biogenesis. IscU pivotal motions would eventually bring the Fe—S cluster biogenesis site on IscU closer to IscS, thus favouring an appropriate coordination distance of the catalytic C328, as observed in the *A*. *fulgidus* crystal structure. No significant correlations of the motions of the two IscU protomers were observed, as they seem to move independently from each other.

While overall the complex is compact, we observe several hints of local dynamics: a *β*-turn of IscU (residues 34–39), which hosts one of the three conserved cysteines involved in the Fe—S cluster coordination (C37), undergoes a local opening. This suggests that such a *β*-turn could play a functional role in iron insertion and cluster formation. Once the cluster is formed, such a motion would be hindered by the coordination of C37, as suggested by the evidence that absence of C37 makes cluster coordination more unstable [43]. The presence of local dynamics strongly suggests that the IscS-IscU interface is somewhat “fluid”, with IscU side chains at the interface being trapped in several local minima, as proven by NMR experiments. The complexity at the interface seems to increase once the ternary IscS-IscU-CyaY complex is formed. This is despite its apparent stability: the ternary complex appears very stable, with CyaY tightly held by the IscS dimer interface through an extended network of salt bridges. Besides the well-characterized arginine triplet (R220, R223 and R225) on IscS, whose replacement has been proven to impair CyaY binding [15], we identified further charged residue pairs constituting a complex salt-bridge network. It will be interesting to test whether and how point-wise or collective mutations of this interface have effects on the complex formation and/or functionality of IscS.

Our data indicate that the presence of CyaY reduces the pivotal motions of the IscU monomers as a whole, as well as the structural fluctuations of the IscS-IscU complex in general, stabilizing opening of the ‘catalytic mouth’ where CyaY is hosted. This is in agreement with the increased affinity of the IscS-IscU assembly when CyaY is bound and may explain mechanistically the cooperativity of this interaction observed experimentally [15]. Over the time scale of our simulation, the flexibility of the IscS catalytic loop in the ternary complex is also almost completely dumped. This is the consequence of a strong and persistent salt bridge between R53 on CyaY and E334 on the IscS catalytic loop, along with steric hindrance. It would be interesting to verify whether the replacement of one of these residues with an alanine or a residue of opposite charge leads to an increase of the mobility of the loop and/or reduction of the binding affinity with CyaY.

The differences we detect between the binary and ternary systems allow us to propose a possible explanation for the molecular mechanism through which CyaY slows down the enzymatic rates of Fe—S cluster formation, according to experimental evidence [19], [44]. We can envisage at least two different but not mutually exclusive ways which could lead to the observed inhibitory effect: the first involves impeding the shuttling motion of the IscS catalytic loop and preventing sulfur delivery to IscU, thereby blocking the biosynthetic route at the stage of the persulfide formation on C328; the second would consist in stabilizing IscU binding within the complex which would reduce the detachment rate. Both mechanisms find additional support in the evidence that the presence of CyaY seems to be incompatible with the arrangement of IscU or of the IscS catalytic loop observed in the *A*. *fulgidus* structure: frataxin orthologues are absent in archaea bacterial species, which pre-dated the evolution of species where frataxin first emerged. It is more difficult to explain mechanistically a role of frataxin as an activator without assuming a different binding mode of this protein in the ternary complex. This is highly unlikely considering the level of conservation of the interface on both IscS and frataxin and the, at least, partial complementation observed with the eukaryotic and prokaryotic proteins [19], [44]. Alternatively, other proteins, such as Isd11, must play an active role in the complex function in eukaryotes.

In conclusion, our results capture for the first time a complex pattern of local dynamical motions within an otherwise stably formed complex. This proves how the overall stability of a protein complex can be well in agreement with local dynamics whose action is crucially relevant for function. We believe that the findings presented here may pave the way for new research and further understanding of the complex molecular mechanism leading to iron-sulfur cluster formation.

## Conflict of interest

The authors declare no conflict of interest.

## Transparency document

Transparency document. Image 2

## Appendix A Supplementary data

Supplementary material Image 2
