# Supplementary material for: Understanding the role of dynamics in the iron sulfur cluster molecular machine
Source: Biochim Biophys Acta. 2017 Jan;1861(1Part A):3154–63. doi: 10.1016/j.bbagen.2016.07.020 (PMC5176006; doi:10.1016/j.bbagen.2016.07.020)
Supplement: Supplementary file 2 — Supplementary material [file mmc1.pdf]

Understanding the molecular origin of frataxin inhibition of Fe-S cluster biosynthesis in prokaryotes

Danilo di Maio, Balasubramanian Chandramouli, Robert Yan, Giuseppe Bancato*, Annalisa Pastore*

Supporting Information

Essential Dynamics Analysis

To further investigate other potential differences between the binary and ternary complexes, we have performed an essential dynamics (ED) analysis on both systems by considering the IscS-IscU tetrameric assembly for the sake of comparison. Specifically, we first performed a PCA on the ternary system and then projected the MD trajectories of the binary and ternary systems onto their respective first two eigenvectors, which in both cases account together for >50% of the total variance. A comparison of the 2D essential subspaces exploration highlights two main differences between the systems considered. Firstly, the ternary complex covers a slightly smaller conformational subspace as compared to the binary system. This further suggests that CyaY binding has a hampering effect on the overall IscS-IscU dynamics. Moreover, the ED plane regions explored by the two systems do not overlap significantly. In principle, this may be due either to an effective difference in the sampling of the same collective motions in the two cases or to a more general and intrinsic difference between the two ED subspaces. The small normalized overlap (~0.261) between the eigenvector sets of the two systems indicates that the large amplitude motions of the two systems are, altogether, quite different. Therefore, we projected the trajectory of the ternary complex onto the eigenvectors of the binary complex in order to better visualize the collective dynamics of the IscS-IscU assembly of the former as compared to the CyaY-free system, e.g. in the motion of the IscS catalytic loop or in the IscU rocking motion. Fig. 7 shows a comparison between such projections onto the ED subspace of the binary system. As a result, the explored conformational subspace of the ternary system is narrower than the binary one, being the dynamics confined within only one "basin" of the ED plane and not at all superimposable with that of the binary system.

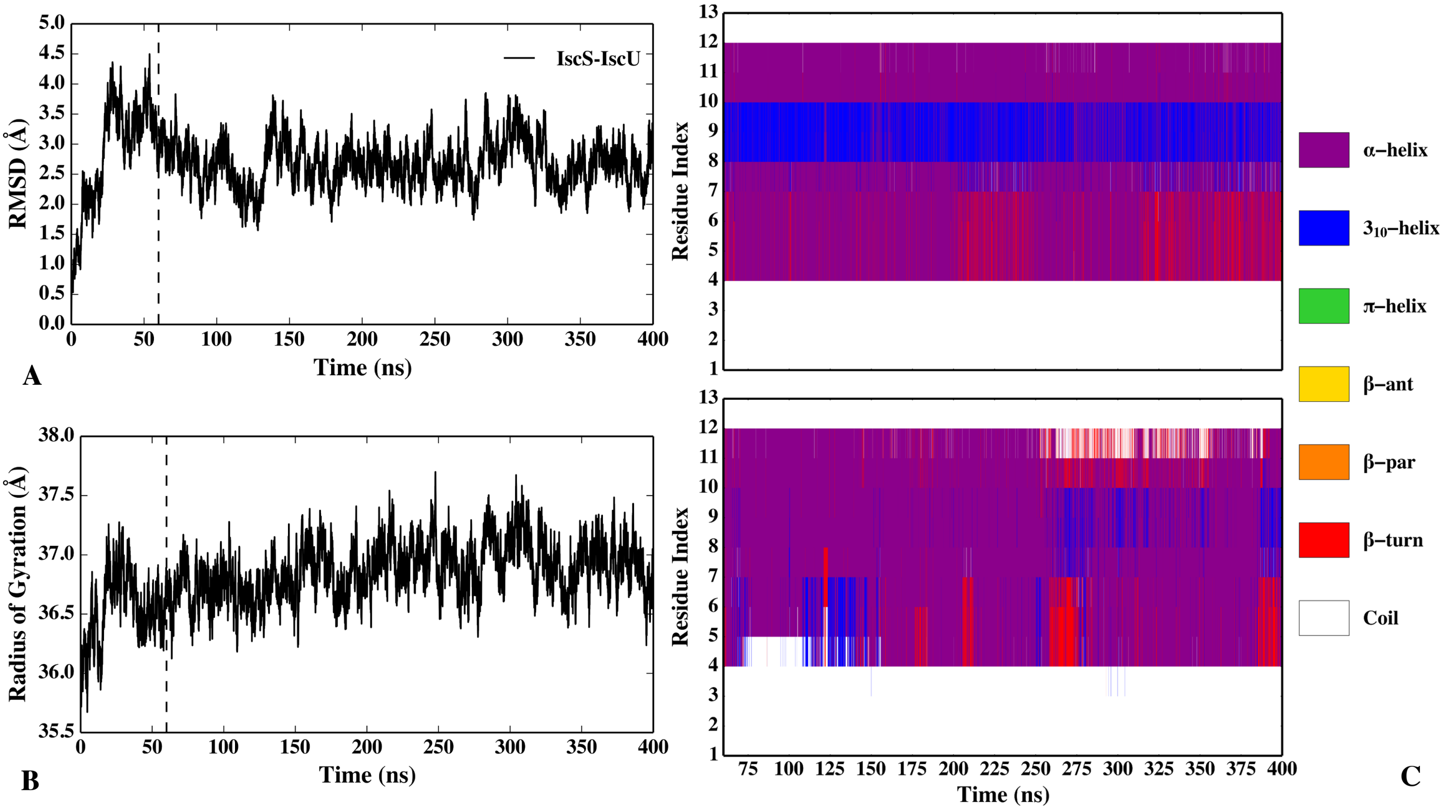

Figure S1. RMSD and radius of gyration of the IscS-IscU complex and analysis of the IscU N-termini. (A) Backbone root-mean-square deviations of the binary system with respect to the starting configuration. Note that, after about 60 ns, RMSD does fluctuate around an average value of approximately 2.6 Å. (B) Radius of gyration of the binary system. (C) Time evolution of the (per-residue) secondary structure content of the N-terminal segment of IscU from both protomers. Note that, despite minor differences between the two N-terminal ends as due to different starting configurations, the average structures are very similar (backbone RMSD = 0.78 Å) and show a high helical content.

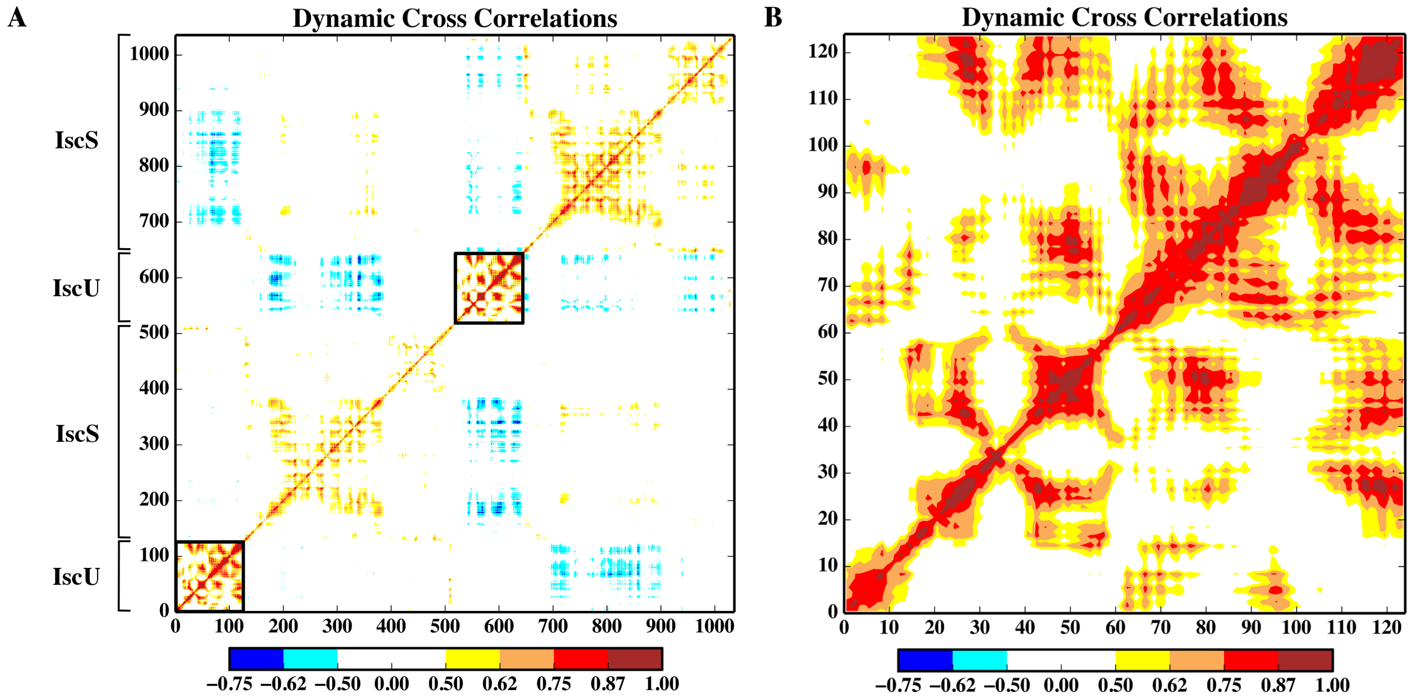

Figure S2. Dynamic cross correlation map. (A) Dynamic residue-residue correlation map of the binary complex shows that the highest correlations (>0.75) are concentrated among the single IscU monomers. (B) Magnified view of the correlation map of one of the two IscU protomers.

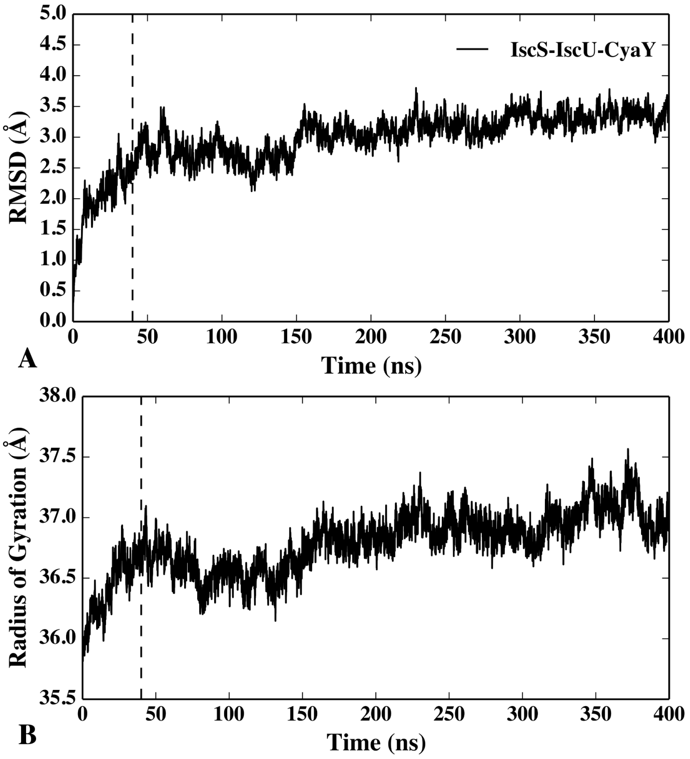

Figure S3. RMSD and radius of gyration of the IscS-IscU-CyaY complex. (A) Backbone root-mean-square deviations of the ternary system with respect to the starting configuration. Note that, after about 40 ns, RMSD does oscillate around an average value of approximately 3 Å. (B) Radius of gyration of the ternary system.

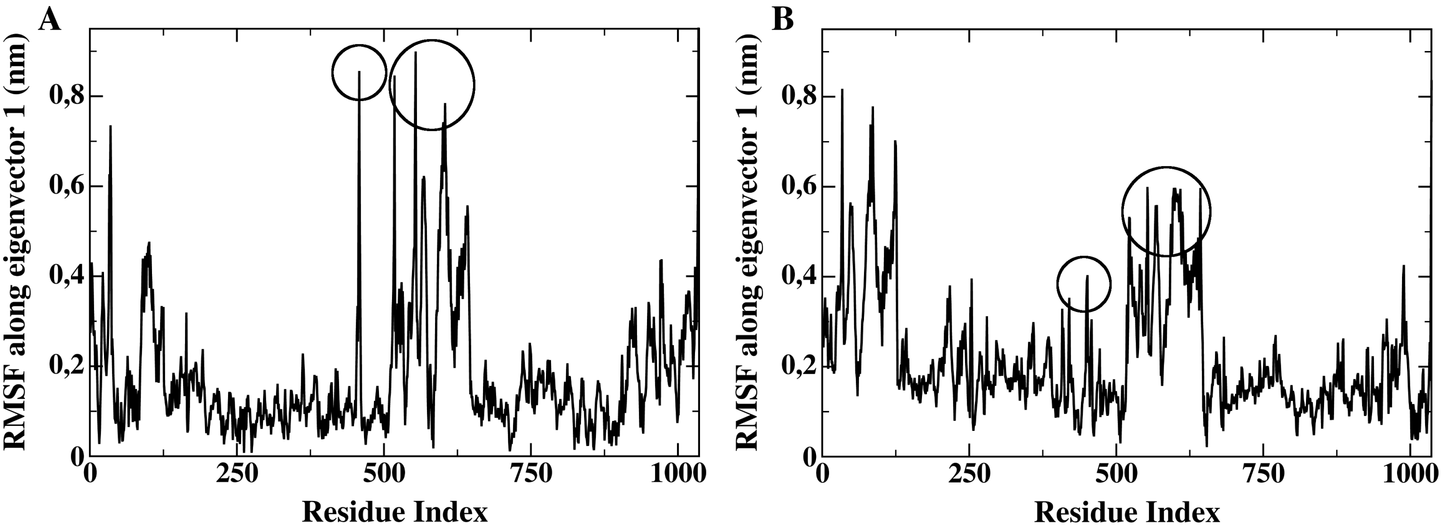

Figure S4. RMSF of the binary and ternary complexes. Per-residue RMSF of (A) the binary and (B) ternary complex along their respective first PCA eigenvector (only IscS-IscU tetramer). Note that in the ternary complex the structural fluctuations are overall hampered: see, for example, the encircled RMSF regions embedding one IscU monomer (residues 519-643 ca) and the IscS catalytic loop (residues 448-462 ca).

Table S1. Salt bridges at the IscS-CyaY interface within the ternary complex. All charged residues (Arg, Lys, Glu, Asp) whose side-chain distance (i.e., N…O) was found below 5 Å were considered in the analysis. The more persistent salt bridges (>35% of total simulation time) are reported, along with the highest percentage observed in both monomers. The analysis revealed no salt bridges between IscU and CyaY.

CyaY	IscS	% Persistence
D11	R112	36.2
D22	R225	92.1
D25	R223	61.5
D25	R220	92.1
D29	R67	91.3
D29	R237	78.9
D29	R220	78.3
D31	R67	85.6
E18	R112	99.0
E19	R116	84.7
E33	R39	97.0
E44	R67	54.8
R53	E334	99.2
R8	E115	75.6
